# Supplementary material for: Recommendations for an exercise intervention and core outcome set for older patients after hospital discharge: Results of an international Delphi study
Source: PLoS One. 2023 Mar 24;18(3):e0283545. doi: 10.1371/journal.pone.0283545 (PMC10038288; doi:10.1371/journal.pone.0283545)
Supplement: S1 Data — (DOCX) [file pone.0283545.s001.docx]

**Supplementary material**

**Screening**

**SCREENING**; measurement tools screening acutely hospitalized older adult after discharge from the hospital

* = Consensus was reached in the first Delphi round; therefore, no median score on Likert-Scale and SIQR were presented.

**Abbreviations**: ABC-scale = Activities-specific Balance Confidence scale; ADL = activities of daily living; BI = Barthel Index; BIS = Bio-Impedance Spectroscopy; CAM = Confusion Assessment Measure; COPM = Canadian Occupational Performance Measure; DOSS = Delirium Observation Screening Scale; EFIP = Evaluation Frailty Index for Physical-activity; EQ-5D = Euro-QoL Health Questionnaire; FAQ = Functional Activities Questionnaire; FES = Fall Efficacy Scale; FSST = Four Square Step Test; GAS = Goal Attainment Scale; GDS = Geriatric Depression Scale; HADS = Hospital Anxiety Depression Scale; HGD = Hand Grip Dynamometry; HHD = Hand Held Dynamometry; IMWG = International Myeloma Working Group; KATZ = Katz-index for independence in activities of daily living; M = median score on Likert-Scale of third Delphi round; MOCA = Montreal Cognitive Assessment; MMSE = Mini Mental State Examination; MNA = Mini Nutritional Assessment; MRC = Medical Research Council; MUST = Malnutrition Universal Screening Tool; NRS = Numeric Rating Scale; PEG-3 = PEG 3-item Scale Assessing Pain Intensity and Interference; PSC = Patient Specific Complaints; QoL = Quality of Life; RM = Repetition Maximum; SIP = Sickness Impact Scale; SIQR = Semi Inter Quartile Range; SF = Short-Form; SNAQ = Short Nutritional Assessment Questionnaire; SPMSQ = Short Portable Mental State Questionnaire; SPPB = Short Physical Performance Battery; VAS = Visual Analogue Scale; 10MWT = 10 meter walk test; 2MWT = 2 minute walk test; 400MWT = 400 meter walking test; 5 times STS = 5 times Sit To Stance

**Requirements in advance of starting an exercise intervention**

* = Consensus was reached in the first Delphi round; therefore, no median score and SIQR were presented.

**Abbreviations:** M = median score on Likert-Scale of third Delphi round. SIQR = semi-inter quartile range.

**Exercise intervention and combined intervention**

**CONDITIONAL ITEMS;** to be able to constitute an appropriate exercise intervention: Personal factors & Interdisciplinary team

**¹** = Acute phase; 5-7 days after acute hospital admission of the older adult

**²** = Subacute phase; 1-12 weeks after acute hospital admission of the older adult

**³** = Longterm phase; >12 weeks after acute hospital admission of the older adult

**Abbreviations:** M = median score on Likert-Scale of third Delphi round, SIQR = semi-inter quartile range.
