# Supplementary material for: Recommendations for an exercise intervention and core outcome set for older patients after hospital discharge: Results of an international Delphi study
Source: PLoS One. 2023 Mar 24;18(3):e0283545. doi: 10.1371/journal.pone.0283545 (PMC10038288; doi:10.1371/journal.pone.0283545)
Supplement: S1 File — (PDF) [file pone.0283545.s002.pdf]

Description of acute hospitalized older adults post-discharge

A ≥ 70 year-old patient who was acutely hospitalized for at least 48 hours. After discharge from the hospital the patient returns to independent living in their own home (not a nursing home or a rehabilitation center).

The population of acutely hospitalized older adults under study does not include patients with complex neurological complications; patients who underwent surgery, patients admitted to the ICU department, patients with a life expectancy of < 3 months, or patients who were admitted to a hospital for surgery in the previous 6 months.

During hospital admission and post-discharge several factors contributing to functional decline are present: consequences of acute disease; immobility; absence of active movement, risk of nutritional deficiency and polypharmacy. It is likely that this patient experiences problems in one or more domains of the ICF. Specific exercise intervention for this target population with a high risk for an increased rate of morbidity, mortality and readmission to a hospital, is not available.

While answering the questions please have in mind the following patient

A 73 year-old patient who was admitted to the hospital five days ago, because of an untreated urinary tract infection, which might be the cause of a delirium. When admitted at the hospital she was treated by a physical therapist twice because of difficulties with getting out of the chair and difficulties with walking. In the beginning the patient was desorientated.

Today the patient is referred from acute treatment setting to his/her own house. One of the still remaining complaints is the continuous lack of energy the patient still experiences.

Before admission to the hospital the patient lived independently in a single-family house, with bed- and bathroom upstairs. The patient lived close to a community center, where spending two evenings a week playing card games. For small grocery shopping the patient went to the supermarket 500 meter from home every other day. Most days the patient walked without a walking aid, but sometimes the patient took walking stick when she was experiencing dizziness (note: 2 fall incidents in previous 6 months; without injury). The oldest daughter came over twice a week to help out with tasks in the household, and make sure the bigger grocery shopping was done.

The general practitioner saw this patient about six times a year, for regulation of the Diabetis Mellitus Type 2 medication and for 2 previous urinary tract infections. In 2017 the patient was admitted to the hospital because of a severe infection in the lungs, after 2 weeks of hospital stay he/she was discharged home. The patient was not admitted to the hospital in the previous six months.
