# Supplementary material for: Recommendations for an exercise intervention and core outcome set for older patients after hospital discharge: Results of an international Delphi study
Source: PLoS One. 2023 Mar 24;18(3):e0283545. doi: 10.1371/journal.pone.0283545 (PMC10038288; doi:10.1371/journal.pone.0283545)
Supplement: Attachment — Submitted filename: Review Plos one paper- FA.docx [file pone.0283545.s003.docx]

23 December 2022

Manuscript review for: Recommendations for an exercise intervention and core outcome set for older patients after hospital discharge: results of an international Delphi study.

Many thanks for the opportunity to review the above-named manuscript. Whilst the aims and objectives for study were clear, I feel there are a few methodological and analytical points of note that may need to be addressed.

My comments are noted below.

**Abstract**

You added the keyword « resistance exercise training ». This term was only used in your abstract! It was not identified neither in the introduction nor in the results « exercise intervention » and figure 2. So, I am confused about the utility of this term. Please clarify

The authors mentioned in their abstract, line 6 “an internal panel of experts n=16” practically only 15 members responded so I think better to mention the number of responders instead.

**Introduction**

Further consideration is needed about this section and the order of the paragraphs and there needs to be a stronger rationale for the research.

Perhaps there needs to give some context to “acute hospitalization” (description and duration). Provide more context about exercise intervention (different types and programs) and its influence on functional decline with few examples and references. You mentioned that practical guidelines on FITT of home-based exercise intervention after hospitalization exists but are lacking! Citing the existing guidelines and giving more details about the content and its limitation could be helpful. Providing this sort would help to set the scene for the study more clearly and give significance to the results.

The authors mentioned in their introduction, line 9 “In this study, in-hospital exercise programs to prevent functional decline were performed twice per day”. Which study? The context is not clear! This part should be addressed.

Further information /details are needed to describe the need for this study.

**Methods**

A stronger narrative on the scoping literature review approach is required.

What were the search keywords used for the scoping literature review?

The search was limited on the last 10 years! Please justify this choice

*Expert Panel.* Selection criteria, homogeneity and panel size (minimum number of experts) were not mentioned in the “expert panel” part. More details are required

Panel members were identified as “experts”, how did you consider or measure experience? Based on years of clinical experience maybe! or number of publications in PubMed? (Cited in table 1) If yes, this should be included in the corresponding paragraph

The authors mentioned in the expert panel part, line 5 “Because no patients were included in this study, approval of the medical committee was not required.” Unsure if this is really useful, I prefer to delete this sentence.

*Delphi rounds.* How expert opinions were collected? Was it based on anonymous survey rounds, face-to-face or group encounters? Please clarify

How did you decide on the number and type of questions?

I would like to have a look on supplementary material on case description and additional questions used in this study.

*Delphi round 3*. The authors mentioned that “If an individual panel member’s scores differed from the panel’s median scores, they were asked to consider re-ranking the statement towards the median to reach consensus.” Not clear. Please explain more the procedure. Was it based on a panel discussion?

**Results**

Why did the authors contact only 16 experts and mainly physical therapists?

*Theme 1.* Daily exercise interventions, line 7 “1–2 times weekly interventions in the sub-acute phase” There is a disagreement with figure 2 where you mentioned that 2-3 times/week is required in sub-acute phase! Please clarify

The panel agreed on level intensity “70-80%” of the maximal heart rate. This requires elaboration about progressive training.

The authors presented the results regarding the consensus but what about the stability of responses between successive rounds? This information would be very interesting to explore

Figure 3 presents measurement tools across ICF domains. How do you explain the choice of these measurement tools knowing that other reliable scales exist in the literature?

**Discussion**

The discussion point about high-intensity exercise interventions. This requires elaboration.

I wonder if the novel finding of this study is “the expert consensus that exercise interventions should be tuned to the specific needs and goals of the patient.” Why was this information not identified in the results part?

Finally, its required to highlight what makes this research novel or unique.
